# Latent Profiles and Influencing Factors of Posttraumatic Stress Symptoms Among Adults During the COVID-19 Pandemic

**DOI:** 10.3389/fpubh.2021.620521

**Published:** 2021-06-24

**Authors:** Wenjie Duan, Qiujie Guan, Qiuping Jin

**Affiliations:** ^1^School of Social and Public Administration, East China University of Science and Technology, Shanghai, China; ^2^School of Social Development and Public Policy, Fudan University, Shanghai, China; ^3^School of Labor and Human Resources, Renmin University of China, Beijing, China

**Keywords:** social media, self-efficacy, COVID-19, latent profile, posttraumatic stress symptoms

## Abstract

The COVID-19 pandemic severely affected public health and the prevalence of posttraumatic stress symptoms among adults in Hubei Province, China. In this study, a total of 2,930 (662 males and 2,268 females) adults answered a questionnaire obtaining information on their demographics, posttraumatic stress symptoms (i.e., intrusion and avoidance), social media exposure, social media involvement, and self-efficacy. Results of the latent profile analysis identified four latent profiles of posttraumatic stress symptoms, which are, no symptoms, high intrusion–low avoidance, moderate symptoms, and high symptoms. The multinomial logistic regression analyses revealed the contributors to the posttraumatic stress symptoms subgroups. Adults with high social media involvement were classified into the high intrusion–low avoidance group, whereas adults with low self-efficacy were included in the moderate symptoms group. Meanwhile, adults with high social media involvement and low self-efficacy were included in the high symptoms group. Interventions may focus on decreasing social media involvement for the adults in the high Intrusion–low avoidance group, improving self-efficacy for the adults in the moderate symptoms group, and reducing social media involvement and improving self-efficacy for the adults in the high symptoms group.

## Introduction

COVID-19 is an infectious disease caused by the most recently discovered coronavirus (1). As global public health threats (2), major infectious diseases can seriously affect public physical health, and cause mental health problems, such as posttraumatic stress symptoms. Posttraumatic stress symptoms refer to a set of mental symptoms triggered by traumatic events (e.g., war, accidents, violence, and disasters) and the experiences of people involved in such events (3), including intrusion, avoidance, hyperarousal, and negative alterations in cognition and mood (4). Major infectious diseases were bio-disasters and traumatic events, which may lead to posttraumatic stress symptoms among wider populations (2). For example, a recent study assessed the prevalence of posttraumatic stress symptoms during coronavirus outbreaks (e.g., SARS, MERS, and COVID-19) through a systematic review and the meta-analysis method and found that posttraumatic stress symptoms are common during coronavirus outbreaks, and approximately one in every 10 individuals from the general population experiences posttraumatic stress symptoms (5). Other empirical studies observed the existence of posttraumatic stress symptoms in the general population during the COVID-19 pandemic. For example, Crosta et al. reported that among 1,253 adults in Italy, approximately 35.59% belong to the high posttraumatic stress symptoms group (6). Liu et al. revealed that 31.8% of young adults in the United States experience high levels of posttraumatic stress symptoms (7). The above studies revealed the prevalence of posttraumatic stress symptoms among adults during the COVID-19 pandemic.

Furthermore, people generally experience different posttraumatic stress symptoms from traumatic events. Specifically, people may exhibit one or more posttraumatic stress symptoms (8), and the severity of each symptom varies (9). This variation indicates the existence of potential posttraumatic stress symptoms profiles among people. Latent profile analysis (LPA) is essential for capturing individual differences. LPA is a person-centered approach that can identify homogeneous subgroups (10), which can be used to develop population-based clinical treatments and interventions. Researchers explored latent posttraumatic stress symptoms profiles in adults with traumatic experiences. For example, Zhou et al. identified three posttraumatic stress symptoms profiles among 191 cancer patients, namely, the non-symptoms group, hyperarousal symptoms group, and severe symptoms group (11). Maguen et al. proposed a four-class posttraumatic stress symptoms profiles for 227 Iraq and Afghanistan veterans, namely, high symptoms, intermediate symptoms, intermediate symptoms with low emotional numbing, and low symptoms (12). However, studies on latent posttraumatic stress symptoms profiles in adults who experienced an infectious disease are limited. In addition, as a novel infectious disease, COVID-19 differs from other infectious diseases in terms of its long incubation period, rapid transmission, and widespread coverage area (13). Thus, using LPA to identify posttraumatic stress symptoms subgroups in adults during the COVID-19 pandemic is necessary to promote the research development of COVID-19.

To reduce the spread of COVID-19, the Chinese government implemented strict “physical distancing and quarantine” measures in the country, especially in Hubei Province. Physical distancing involves reducing close physical contact, and quarantine means restricting public activities or segregating individuals who are well but may have been exposed to COVID-19 (14). Although physical distancing and quarantine entail physical separation, social connections persist through social media platforms (15). Previous studies revealed the “double-edged sword” role of social media. On the one hand, social media can help ease anxiety and increase positive emotions during the COVID-19 pandemic (16). On the other hand, using social media to obtain information on COVID-19 may amplify the threats of the disease and cause mental health problems (17). In the use of social media, social media exposure and involvement play a significant role in the prevalence of posttraumatic stress symptoms. Social media exposure refers to people's active or passive collection of information about COVID-19 from social media (18), whereas social media involvement refers to people's attention to and participation in social media (19), such as sharing and posting information about COVID-19. A recent study reported that in 4,827 Chinese adults, over 80% report frequent exposure to news and information about COVID-19 on social media (20). In terms of the impact of posttraumatic stress symptoms, previous studies examined the contribution of social media use to posttraumatic stress symptoms. For example, a study on 967 adults showed that compared with direct exposure to Hurricane Sandy, using social media to learn about Hurricane Sandy can cause posttraumatic stress symptoms (21). Monfort and Afzali investigated the posttraumatic stress symptoms experienced by 451 young adults after the 2015 terrorist attack in Paris and found that social media use is a predictor of posttraumatic stress symptoms (22). However, the impact of social media exposure and involvement on posttraumatic stress symptoms should be proven.

During physical distancing and quarantine periods, people's self-efficacy is closely related to posttraumatic stress symptoms. Self-efficacy is a positive personality characteristic that refers to an individuals' belief in his/her ability to execute or accomplish a task (23). Individuals with a high level of self-efficacy typically have positive mental health and a low likelihood of experiencing posttraumatic stress symptoms. For example, Nygaard et al. surveyed 617 adults who experienced the 2004 Southeast Asian tsunami and revealed a negative relationship between self-efficacy and posttraumatic stress symptoms (24). Meanwhile, LeBlanc found that people who perceive a low level of self-efficacy exhibit posttraumatic stress symptoms (25). Thus, self-efficacy may be a predictor of posttraumatic stress symptoms among individuals during the COVID-19 pandemic.

Based on existing research results, speculating that adults in Hubei Province may have different posttraumatic stress symptoms profiles during the COVID-19 pandemic is reasonable. Moreover, social media exposure, social media involvement, and self-efficacy may predict latent posttraumatic stress symptoms profiles. Considering intrusion and avoidance as core and basic posttraumatic stress symptoms, the present study focuses on the latent profiles of intrusion and avoidance (26). In summary, this study aims to (a) identify latent profiles of intrusion and avoidance among adults in Hubei Province and (b) explore whether social media exposure, social media involvement, and self-efficacy are contributors to different profiles of intrusion and avoidance.

## Method

### Participants and Procedure

The sample in this study was a subset in the Social Cognition and Behavior Investigation of COVID-19 survey. This survey was conducted from January 31 to February 8, 2020, which was the peak of the COVID-19 outbreak in Mainland China. The survey aimed to understand how people in Wuhan; other cities in Hubei, excluding Wuhan; and other cities outside Hubei perceived and responded to COVID-19. The characteristics of COVID-19 (13) make most individuals without protection susceptible to infection. Participants were recruited via convenience sampling through social media. Convenience sampling through social media is a typical and common method used in public health emergency studies (27, 28). A total of 7,058 individuals (2,157 males and 4,901 females; mean age = 26.06 years, SD = 12.91, range = 8–72 years) participated voluntarily in the investigation. Ethics approval was obtained from the Human Subjects Ethics Sub-Committee of [anonymous for peer review]. The participants clicked on the agree button to indicate their agreement and informed consent before completing the questionnaire.

The participants of the current study (a) were residents of Hubei Province, (b) were over 18 years old, (c) could complete the online survey through social media, (d) could understand Chinese, and e) considered COVID-19 as a major stressful event in the past 2 weeks. Specifically, a criterion for the participants who considered COVID-19 as a major stressful event was that they perceived threat and stress from COVID-19 in the past 2 weeks, including the items “*My family/friends/neighbors and I may be infected with COVID-19*” (perceived threat) and “*I feel stressed about COVID-19*” (perceived stress). Participants who claimed to be positive, suspected to be positive, or survived the disease were excluded. Ultimately, 2,930 adults participated in the current study, including 662 males (mean age = 39.98 years, SD = 7.18) and 2,268 females (mean age = 37.12 years, SD = 6.42).

Table 1 presents the demographic information of the participants. Among the participants, 66.28% (*N* = 1,942) attained a high school education or above. The subjective socioeconomic status of the participants was measured using the MacArthur Scale of Subjective Socioeconomic Status Ladder (29), with 10 rungs ranging from 1 (lowest) to −10 (highest). In addition, 34.03% of the participants (*N* = 997) indicated having a middle socioeconomic status. For the self-reported general health, the participants were required to rate their general health as “very poor,” “poor,” “normal,” “good,” or “very good,” and approximately 74.95% of the participants (*N* = 2,196) reported having “good” or “very good” health.

**Table 1:** Descriptive statistics of main variables and sample characteristics (*N* = 2,930).

**Variables**	**N**	**Percentage**
	***Mean* ±*SD***	**Range**
Total posttraumatic stress symptoms	16.96 ± 7.88	0–40
Intrusion	10.46 ± 5.04	0–20
Avoidance	6.51 ± 4.52	0–20
Social media exposure	5.00 ± 1.19	1–6
Social media involvement	3.51 ± 1.71	1–6
Self-efficacy	3.79 ± 0.71	1–5
Gender		
Male	662	22.59%
Female	2,268	77.41%
Educational level		
Primary school and below	172	5.87%
Junior school	816	27.85%
High school	889	30.34%
Bachelor and above	1,053	35.94%
Subjective socioeconomic status		
1 (lowest)	217	7.41%
2	105	3.58%
3	232	7.92%
4	257	8.77%
5	997	34.03%
6	634	21.64%
7	280	9.56%
8	164	5.60%
9	21	0.72%
10 (highest)	23	0.78%
Self-reported general health		
Very poor	4	0.14%
Poor	44	1.50%
Normal	686	23.41%
Good	1,366	46.62%
Very good	830	28.33%

### Measures

#### Posttraumatic Stress Symptoms

Posttraumatic stress symptoms were measured by an eight-item version of the Impact of Event Scale, which is a short version of the original 15-item scale (30). The eight-item version of the Impact of Event Scale contained two subscales, namely, intrusion and avoidance (31), which comprised four items each. The keywords for the items were modified to suit the current situation (e.g., “*Try to remove it from my memory*” was changed to “*Try to remove COVID-19 from my memory*”) (32). The participants were required to answer the questions using a four-point Likert scale (0 = not at all, 1 = rarely, 3 = sometimes, 5 = often). The total score of each subscale represented the score of each dimension. The scale demonstrated good internal consistency coefficients (Cronbach's alpha = 0.78) in the previous study (31). In the current study, the Cronbach's alpha vales of the entire scale, intrusion subscale, and avoidance subscale were above 0.82.

#### Social Media Exposure and Involvement

Two items were developed to assess social media exposure and involvement based on a previous study on MERS (33). One item (i.e., frequency of seeing or hearing information about COVID-19 on social media) was used to assess social media exposure, and the participants were required to answer the question on a six-point scale (ranging from 1 = rarely to 6 = always). The higher the score, the more the social media exposure. Social media involvement was measured by the other item (i.e., frequency of posting or sharing information about COVID-19 on social media), and participants were instructed to answer the question on a six-point scale (ranging from 1 = rarely to 6 = always). The higher the score, the more the social media involvement.

#### Self-efficacy

Self-efficacy in terms of COVID-19 was assessed with a four-item scale adopted from previous studies (33, 34). The respondents were asked to indicate the extent to which they agreed or disagreed with the statements about their self-efficacy on a five-point Likert scale ranging from 1 (strongly disagree) to 5 (strongly agree). The keywords were modified based on the current pandemic. High mean scores indicate high levels of self-efficacy in terms of COVID-19. The scale was reliable, with a Cronbach's alpha score of 0.78 in the previous study (33). In the present study, the Cronbach's alpha of the scale was 0.71.

### Data Analysis

First, the descriptive and correlation statistics of the main variables (i.e., total posttraumatic stress symptoms, intrusion, avoidance, social media exposure, social media involvement, and self-efficacy) were obtained. Second, LPA was conducted to determine the latent profiles of intrusion and avoidance based on the scores of the eight items. LPA is a person-oriented approach that exhibits advantages over variable-oriented approaches. Variable-oriented approaches are used to identify variables of interest and describe their relations with individuals (35), whereas LPA focuses on identifying common attributes at the individual level and distinguishing homogeneous subgroups (10). The following indices were employed to determine the fitness of the results: the low Akaike information criteria (AIC), Bayesian information criterion (BIC), adjusted BIC values (ABIC), high entropy, and a significant value (*p* < 0.001) of Lo–Mendell–Rubin and likelihood ratio test (LMR-LRT), which indicates a superior fit (36). Third, multivariate ANOVA was conducted to test the group differences in the main variables. Finally, multinomial logistic regression analyses were performed to examine the association between the latent profiles of intrusion and avoidance and contributors (i.e., social media exposure, social media involvement, and self-efficacy). The data were analyzed using SPSS 24.0 and Mplus 7.4.

## Results

### Descriptive and Correlation Statistics

The descriptive statistics (*mean* ± *SD*) of the main variables are presented in Table 1. For the correlations among the variables, total posttraumatic stress symptoms was positively related to social media exposure (*r* = 0.06, *p* < 0.01) and social media involvement (*r* = 0.14, *p* < 0.01), but negatively related to self-efficacy (*r* = −0.04, *p* < 0.05). Intrusion was positively correlated with avoidance (*r* = 0.36, *p* < 0.01), social media exposure (*r* = 0.12, *p* < 0.01), and involvement (*r* = 0.17, *p* < 0.01), whereas avoidance was negative related to self-efficacy (*r* = −0.06, *p* < 0.01).

### Latent Profile Analysis

Table 2 displays the relevant indices of the LPA results. Based on the LMR-LRT, the two-to five- profile solutions were acceptable. The five-profile solution was rejected because it included a subgroup comprising <10% of the total sample. Given that the BIC was the most sensitive LPA index (36), the four-profile solution was the fittest.

**Table 2:** Model fit indexes of latent profile analysis (*N* = 2,930).

**Model**	**AIC**	**BIC**	**ABIC**	**Entropy**	**LMR *P*-value**	**LRT *P*-value**	**Minimum Class Size *N* (%)**
Two-profile	54108.81	54258.37	54178.94	0.84	<0.0001	<0.0001	1,124 (38.36%)
Three-profile	51978.99	52182.41	52074.38	0.86	<0.0001	<0.0001	412 (14.06%)
**Four-profile**	**50587.92**	**50845.18**	**50708.56**	**0.85**	** <0.0001**	** <0.0001**	**396 (13.52%)**
Five-profile	49972.34	50283.44	50118.22	0.86	<0.0001	<0.0001	233 (7.95%)
Six-profile	49795.77	50160.72	49966.89	0.86	0.3012	0.2958	90 (3.07%)

*AIC, Akaike's information criterion; BIC, Bayesian Information Criterion; ABIC, Sample-size adjusted BIC; LMR, Lo-Mendell-Rubin adjusted likelihood ratio test; LRT, Bootstrapped likelihood ratio test. Bold represents best fit for each respective statistic*.

**Figure 1:**
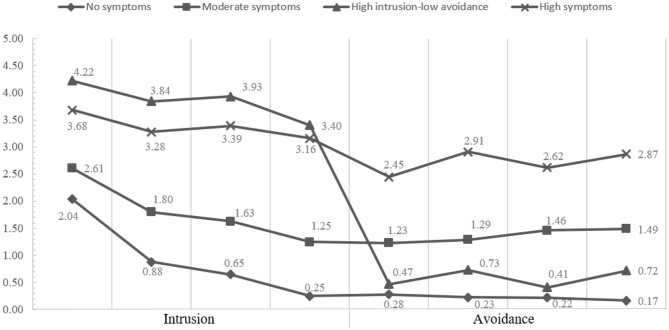
Standardized means of intrusion and avoidance across four profiles (*N* = 2,930).

Profile 1 included 13.52% of the total sample (*N* = 396) and representative participants without posttraumatic stress symptoms (no symptoms group). Profile 2 comprised 14.71% of the total sample (*N* = 431) and representative participants with high levels of intrusion and low levels of avoidance (high intrusion–low avoidance group). Profile 3 included 32.56% of the total sample (*N* = 954, and representative participants with moderate levels of intrusion and avoidance (moderate symptoms group). Profile 4 consisted of 39.21% of the total sample (*N* = 1,149) and representative participants with high levels of intrusion and avoidance (high symptoms group). The standardized means of the four profiles are presented in Figure 1.

### Multivariate ANOVA Analysis

The ANOVA indicated that the four groups (i.e., no symptoms group, high intrusion–low avoidance group, moderate symptoms group, high symptoms group) exhibited significant differences in terms of the total posttraumatic stress symptoms (*F* = 3212.09, *p* < 0.001), intrusion (*F* = 1812.57, *p* < 0.001), and avoidance (*F* = 2448.35, *p* < 0.001). The results also showed significant differences in the four groups in social media exposure (*F* = 6.13, *p* < 0.001), social media involvement (*F* = 18.88, *p* < 0.001), and self-efficacy (*F* = 8.08, *p* < 0.001). Specifically, the participants in the no symptoms group demonstrated high levels of self-efficacy (mean = 3.89, *SD* = 0.68) and low levels of social media exposure (mean = 4.97, *SD* = 1.26) and social media involvement (mean = 3.25, *SD* = 1.74). The participants in the high intrusion–low avoidance group obtained high scores on social media exposure (mean = 5.20, *SD* = 1.12), social media involvement (mean = 3.86, *SD* = 1.65), and self-efficacy (mean = 3.90, *SD* = 0.77). The participants in the moderate symptoms group scored low on social media exposure (mean = 4.91, *SD* = 1.20), social media involvement (mean = 3.27, *SD* = 1.71), and self-efficacy (mean = 3.75, *SD* = 0.68). Finally, the participants in the high symptoms group showed high levels of social media exposure (mean = 5.02, *SD* = 1.18) and social media involvement (mean = 3.66, *SD* = 1.69) and low levels of self-efficacy (mean = 3.75, *SD* = 0.70).

### Multinomial Logistic Regression Analyses

The high intrusion–low avoidance, moderate symptoms, and high symptoms groups were compared with the no symptoms group as the reference group. Table 3 shows that compared with the no symptoms group, (a) the adults with high social media involvement (OR = 1.21, 95%CI = 1.11–1.32) were classified into the High Intrusion-Low Avoidance group, (b) the adults with low self-efficacy (OR = 0.76, 95% CI = 0.64–0.90) had a high probability of being classified into the moderate symptoms group, and (c) the adults who reported high social media involvement (OR = 1.18, 95%CI = 1.09–1.26) and low self-efficacy (OR = 0.73, 95%CI = 0.62–0.87) were placed in the high symptoms group. However, social media exposure had no influence on the three symptoms groups.

**Table 3:** Multinomial logistic regression modeling results of four profiles (*N* = 2,930).

	***B***	**SE**	***p***	**Odds Ratio**	**95% CI for Odds Ratio**
**High intrusion–low avoidance vs. No symptoms**					
Social media exposure	0.07	0.07	0.27	1.08	[0.95, 1.22]
Social media involvement	0.19	0.04	**0.00**	1.21	[1.11, 1.32]
Self-efficacy	−0.03	0.10	0.75	0.97	[0.79, 1.18]
**Moderate symptoms vs. No symptoms**					
Social media exposure	−0.04	0.05	0.44	0.96	[0.87, 1.06]
Social media involvement	0.03	0.04	0.50	1.03	[0.95, 1.10]
Self-efficacy	−0.28	0.09	**0.001**	0.76	[0.64, 0.90]
**High symptoms vs. No symptoms**					
Social media exposure	−0.03	0.05	0.55	0.97	[0.88, 1.07]
Social media involvement	0.16	0.04	**0.00**	1.18	[1.09, 1.26]
Self-efficacy	−0.31	0.09	**0.00**	0.73	[0.62, 0.87]

*CI, confidence interval. The influences for statistical significant are in bold*.

Furthermore, the no symptoms, moderate symptoms, and high symptoms groups were compared with the high intrusion–low avoidance group as the reference group. The results revealed that (a) the adults with low social media involvement (OR = 0.84, 95% CI = 0.76–0.90) were classified into the no symptoms group; (b) the adults with low social media exposure (OR = 0.89, 95% CI = 0.80–0.99), social media involvement (OR = 0.85, 95% CI = 0.79–0.91), and self-efficacy (OR = 0.78, 95% CI = 0.66–0.92) had a high probability of being included in the moderate symptoms group; and (c) the adults who reported low self-efficacy (OR = 0.76, 95% CI = 0.64–0.89) were designated to the high symptoms group.

## Discussion

The current study explored the latent profiles of posttraumatic stress symptoms (i.e., intrusion and avoidance) among adults in Hubei Province during the COVID-19 pandemic. The results identified a four-profile solution that included a no symptoms group, high intrusion–low avoidance group, moderate symptoms group, and high symptoms group. The results of the multinomial logistic regression analyses validated the contribution of social media involvement and self-efficacy to the subgroups. Specifically, high social media involvement contributed to high intrusion and low avoidance levels, low self-efficacy contributed to moderate symptoms, and high social media involvement and low self-efficacy were associated with high symptoms. Ultimately, social media exposure showed no influence on the latent profiles of intrusion and avoidance.

The no symptoms, moderate symptoms, and high symptoms groups identified in the current study were similar to the subgroups among adults who experienced other traumatic events. For example, a study explored latent posttraumatic stress symptoms classes in 810 adults during a hurricane and identified a four-class pattern comprising severe, moderate, mild, and negligible groups (37). However, the high intrusion–low avoidance group that emerged in this study differed from existing posttraumatic stress symptoms groups. Thus, discussing the differences between the high intrusion–low avoidance group and high symptoms group is essential and meaningful. On the one hand, the participants in the high intrusion–low avoidance group demonstrated intrusion, whereas the participants in the high symptoms group exhibited intrusion and avoidance. On the other hand, the results of the ANOVA revealed that the adults in the high intrusion–low avoidance group had high levels of social media involvement and self-efficacy, whereas the adults in the high symptoms group had high levels of social media involvement and low levels of self-efficacy. The above findings indicated that self-efficacy may be a predictor of low avoidance. The results of the correlation analysis also provided evidence for the negative relationship between avoidance and self-efficacy. Thus, self-efficacy improvement can be used in interventions to reduce avoidance.

The present study focused on social media exposure to and involvement in COVID-19 information and determined the predictable role of social media involvement in posttraumatic stress symptoms. However, social media exposure exerted no influence on posttraumatic stress symptoms, which was inconsistent with our primary hypothesis. Social media exposure and involvement had different meanings in the current study. Social media exposure refers to people actively or passively obtaining information (i.e., seeing or hearing information) about COVID-19 from social media (18). Meanwhile, social media involvement refers to the behavior of actively obtaining information (i.e., posting, sharing, and commenting on information) about COVID-19 from social media, which entails increased attention to and engagement in information about COVID-19 (19). Moreover, social media exposure and social media involvement refer to the varying degrees that people indulge in social media (38). Social media exposure emphasizes receiving information about COVID-19, whereas social media involvement involves receiving and sharing information about COVID-19. Thus, social media involvement entails more active behaviors and higher indulgence than social media exposure. Furthermore, social media exposure and involvement exert different influences on posttraumatic stress symptoms. With the popularity of social media and diversification of its functions, social media exposure to COVID-19 information is common (20). All social media users can receive information about COVID-19, which may be why social media exposure had an insignificant impact on posttraumatic stress symptoms. In addition, as mentioned above, social media involvement indicates deeper indulgence in social media than social media exposure. Studies pointed out that high social media involvement may amplify adults' perceived risks of COVID-19 (17), which may harm public mental health. Therefore, in our study social media involvement showing a significant influence on posttraumatic stress symptoms is understandable. Overall, the results highlighted the significant role of social media involvement and self-efficacy and provided evidence for population-based clinical treatments and interventions. For the high intrusion–low avoidance group, interventions should aim to reduce social media involvement (e.g., decrease time spent on social media). For the moderate symptoms group, interventions based on self-efficacy may be effective to reduce posttraumatic stress symptoms in adults (e.g., improve belief in ability to overcome COVID-19). For the high symptoms group, social media involvement and self-efficacy may be essential for interventions.

However, several limitations and directions for future research should be noted. First, the sample was unevenly distributed, which may influence the results. To determine whether the findings can be applied to a demographically representative sample, a subsample (*N* = 1,063) was created by randomly reducing the data to match the census records in terms of gender (male vs. female) and age (ranging from 35 years to 54 years). The census data of Hubei Province were obtained from reports by the National Bureau of Statistics (39). Similar results were observed in the demographically representative sample (see the Supplementary Documents). Fundamentally, researchers should consider using highly efficient methods in the future to address the issue of representativeness. Second, the current scale assessed limited posttraumatic stress symptoms (i.e., intrusion and avoidance). Thus, other symptoms (e.g., hyperarousal and negative alterations in cognition and mood) should be examined, and the latest multidimensional tools should be employed in future studies. The third issue concerns the cross-cultural applicability of the eight-item version of the Impact of Event Scale. Actually, the original 15-item version of the Impact of Event Scale was previously validated in the Western contexts (40) and the Chinese contexts (41), which showed satisfactory psychometric characteristics among adults. Therefore, we believe that the short version of the Impact of Event Scale used in the current study may also have cross-cultural applicability. Finally, data were collected using a cross-sectional design, but a longitudinal study should be conducted to further examine the characteristics of posttraumatic stress symptoms in adults.

In conclusion, this study targeted adults in Hubei Province, China, to investigate the heterogeneity of posttraumatic stress symptoms (i.e., intrusion and avoidance) and examine the factors contributing to posttraumatic stress symptoms subgroups during the COVID-19 pandemic. The results showed that social media involvement and self-efficacy may be predictors of posttraumatic stress symptoms among adults in Hubei Province. The findings provided evidence for public health management during the COVID-19 pandemic. On the one hand, social media plays a significant role in disseminating risk information on COVID-19. However, social media involvement may amplify adults' perceived risks of COVID-19 (17) and threaten their mental health. Thus, scientific media broadcasts and moderate social media involvement should be promoted in public health management. On the other hand, interventions promoting self-efficacy should be implemented widely by social workers and psychologists to help improve public health.

## Data Availability Statement

The raw data supporting the conclusions of this article will be made available by the authors, without undue reservation.

## Author Contributions

WD: conceptualization, methodology, visualization, writing—review and editing, supervision, and project administration. QG: conceptualization, methodology, formal analysis, and writing—original draft. QJ: conceptualization, methodology, visualization, and writing—review and editing. All authors contributed to the article and approved the submitted version.

## Conflict of Interest

The authors declare that the research was conducted in the absence of any commercial or financial relationships that could be construed as a potential conflict of interest.
