# Supplementary material for: Latent Profiles and Influencing Factors of Posttraumatic Stress Symptoms Among Adults During the COVID-19 Pandemic
Source: Front Public Health. 2021 Jun 24;9:620521. doi: 10.3389/fpubh.2021.620521 (PMC8264580; doi:10.3389/fpubh.2021.620521)
Supplement: Supplementary file 1 [file Data_Sheet_1.pdf]

Supplement Materials: Details about analyses of a demographically representative sample

A subsample (N = 1,063) was created and analyzed through randomly reducing the data to match the census records in important dimensions of demographics, namely, sex (male vs. female) and age (range: 35-54). The census data of Hubei Province were obtained from reports by the National Bureau of Statistics (2011).

Similar results were observed in this demographically-representative sample. Specifically, the results of latent profile analysis identified four latent profiles of intrusion and avoidance, which were no symptoms, high intrusion-low avoidance, moderate symptoms, and high symptoms. Multinomial logistic regression analyses indicated contributors of different subgroups. Adults with more social media involvement were classified to high intrusion-low avoidance group. Less self-efficacy of adults probably being a member of moderate symptoms group. Adults reported more social media involvement and less self-efficacy were distributed to the high symptoms group. Detailed information are as follows.

Sample characteristics.

Table S1 shows the sample demographic information of participants. A total of 1,063 adults (mean age = 41.13, SD = 4.18) involved in the current study, including 521 males and 542 females. Among them, 67.8% participants (N = 721) received high school or above education. The subjective socioeconomic status of participants was measured using the MacArthur Scale of Subjective Socioeconomic Status ladder (Adler et al., 2000), the ladder with 10 rungs ranging from 1 (lowest) to 10 (highest). A total of 31.9% participants (N = 339) chose middle socioeconomic status. For self-reported general health, participants were required to rate their general health as one of very poor, poor, normal, good, and very good, about 73.3% participants (N = 779) reported good and very good health.

Descriptive and Correlation Statistics.

The descriptive statistics (Mean ± SD) of the main variables are presented in Table 1. The total posttraumatic stress symptoms was positively related to social media exposure ($r = .07$, $p < .05$), social media involvement ($r = .18$, $p < .01$). Intrusion was positively correlated with avoidance ($r = .36$, $p < .01$),

social media exposure ($r = .11$, $p < .01$) and involvement ($r = .22$, $p < .01$). Avoidance was negative related to self-efficacy ($r = -.08$, $p < .05$).

Latent Profile Analysis

Table S2 displays necessary indices of the LPA results. The five-profile solution was rejected because it contained a subgroup covering less than 10% total samples and the *p*-value of LMR was insignificant. Given that BIC was the most sensitive index for LPA (Nylund et al., 2007), the four-profile solution was the fittest.

Profile 1 contained 12.9% of total sample (N = 137), and representative participants with no symptoms (no symptoms group). Profile 2 contained 14.9% of total sample (N = 158), and representative participants showed high levels of intrusion and low levels of avoidance (high intrusion-low avoidance group). Profile 3 contained 34.3% of total sample (N = 365), and representative participants showed moderate intrusion and avoidance (moderate symptoms group). Profile 4 contained 37.9% of total sample (N = 403), and representative participants showed high levels of both intrusion and avoidance (high symptoms group). Standardized means of the four profiles was available in Fig. S1.

Multivariate ANOVA Analysis

The analysis of variance indicated the four groups (i.e., no symptoms group, high intrusion-low avoidance group, moderate symptoms group, high symptoms group) showed significant difference from each other on the total posttraumatic stress symptoms ($F = 1114.54$, $p < .001$), intrusion ($F = 670.16$, $p < .001$), and avoidance ($F = 836.82$, $p < .001$). Results also showed significant difference on social media involvement ($F = 8.20$, $p < .001$) and self-efficacy ($F = 5.56$, $p < .001$) among the four groups. Specifically, participants in high intrusion-low avoidance group had the highest scores on social media involvement (mean = 3.89, SD = 1.63). Participants in the high symptoms group scored lowest on self-efficacy (mean = 3.75, SD = 0.67). Participants in the no symptoms group showed highest level of self-efficacy (mean = 3.97, SD = 0.63) and lowest level of social media involvement (mean = 3.21, SD = 1.76). However, the four group showed no significant differences on social media exposure.

Multinomial Logistic Regression Analyses

Taking the no symptoms group as the reference group, the high intrusion-low avoidance group, the moderate symptoms group, and the high symptoms group were compared with it. As shown in Table S3, compared with the no symptoms group, (a) adults with more social media involvement (OR = 1.27, 95%CI = 1.09-1.47) were classified into the high intrusion-low avoidance group, (b) the lower self-efficacy (OR = 0.62, 95%CI = 0.46-0.84), the higher probability of being a member of the moderate symptoms group, (c) adults who reported more social media involvement (OR = 1.18, 95%CI = 1.05-1.34) and less self-efficacy (OR = 0.59, 95%CI = 0.44-0.80) were distributed to the high symptoms group. However, social media exposure has no influence on the three groups.

Furthermore, taking the high intrusion-low avoidance group as the reference group, the no symptoms group, the moderate symptoms group, and the high symptoms group were compared with it. Results found that (a) adults with less social media involvement (OR = 0.79, 95%CI = 0.68-0.92) were classified into the no symptoms group, (b) the less social media involvement (OR = 0.81, 95%CI = 0.72-0.91) and self-efficacy (OR = 0.74, 95%CI = 0.56-0.97), the higher probability of being a member of the moderate symptoms group, (c) adults who reported less self-efficacy (OR = 0.70, 95%CI = 0.53-0.92) were distributed to the high symptoms group.

Table S1. *Descriptive statistics of the main variables and sample characteristics.*

	Current sample (N = 1,063)		Census records of Hubei Province	
	N<br>M ± SD	%<br>Range	N<br>M ± SD	%<br>Range
The total posttraumatic stress symptoms	16.69 ± 7.84	0-40		
Intrusion	10.25 ± 5.04	0-20		
Avoidance	6.44 ± 4.46	0-20		
Social media exposure	4.95 ± 1.19	1-6		
Social media involvement	3.51 ± 1.71	1-6		
Self-efficacy	3.80 ± 0.69	1-5		
Age	41.13 ± 4.18	35-54	/	35-54
Gender				
Male	521	49.00%	9832888	49.00%
Female	542	51.00%	9449070	51.00%
Education level				
Primary school and below	72	6.8%	/	/
Junior school	270	25.4%	/	/
High school	337	31.7%	/	/
Bachelor and above	384	36.1%	/	/
Subjective socioeconomic status				
1 (Lowest)	94	8.8%	/	/
2	44	4.1%	/	/
3	92	8.7%	/	/
4	77	7.2%	/	/
5	339	31.9%	/	/
6	244	23.0%	/	/
7	111	10.4%	/	/
8	50	4.7%	/	/
9	10	0.9%	/	/
10 (Highest)	2	0.2%	/	/
Self-reported general health				
Very poor	0	0.0%	/	/

	Poor	18	1.7%	/	/
	Normal	266	25.0%	/	/
	Good	480	45.2%	/	/
	Very good	299	28.1%	/	/

Table S2. *Model fit indexes of latent profile analysis (N = 1,063)*

Model	AIC	BIC	ABIC	Entropy	LMR *P*-value	LRT *P*-value	Minimum Class Size N (%)
2-profile	19532.11	19656.33	19576.92	0.84	< 0.0001	< 0.0001	405 (38.12%)
3-profile	18767.76	18936.70	18828.71	0.85	< 0.0001	< 0.0001	151 (14.06%)
**4- profile**	**18154.71**	**18368.37**	**18231.79**	**0.86**	**0.0009**	**< 0.0001**	**137 (12.89%)**
5- profile	17917.79	18176.17	18011.01	0.88	0.0621	< 0.0001	76 (7.15%)

Note. AIC = Akaike's information criterion; BIC = Bayesian Information Criterion; ABIC = Sample-size adjusted BIC; LMR = Lo-Mendell-Rubin adjusted likelihood ratio test; LRT = bootstrapped likelihood ratio test.

Table S3. *Multinomial logistic regression modelling results for the four profiles (N = 1,063)*

	B	SE	*p*	Odds Ratio	95% CI for Odds Ratio
**High intrusion-low avoidance group vs. No symptoms group**					
Social media exposure	0.01	0.11	0.89	1.02	[0.82, 1.25]
Social media involvement	0.23	0.08	**0.002**	1.27	[1.09, 1.47]
Self-efficacy	-0.17	0.18	0.34	0.84	[0.60, 1.19]
**Moderate symptoms group vs. No symptoms group**					
Social Media Exposure	0.03	0.09	0.73	1.03	[0.87, 1.22]
Social Media Involvement	0.03	0.06	0.69	1.03	[0.91, 1.16]
Self-efficacy	-0.48	0.15	**0.002**	0.62	[0.46, 0.84]
**High symptoms group vs. No symptoms group**					
Social Media Exposure	0.06	0.09	0.50	1.06	[0.89, 1.26]
Social Media Involvement	0.17	0.06	**0.008**	1.18	[1.05, 1.34]
Self-efficacy	-0.52	0.15	**0.000**	0.59	[0.44, 0.80]

Note. CI = Confidence Interval

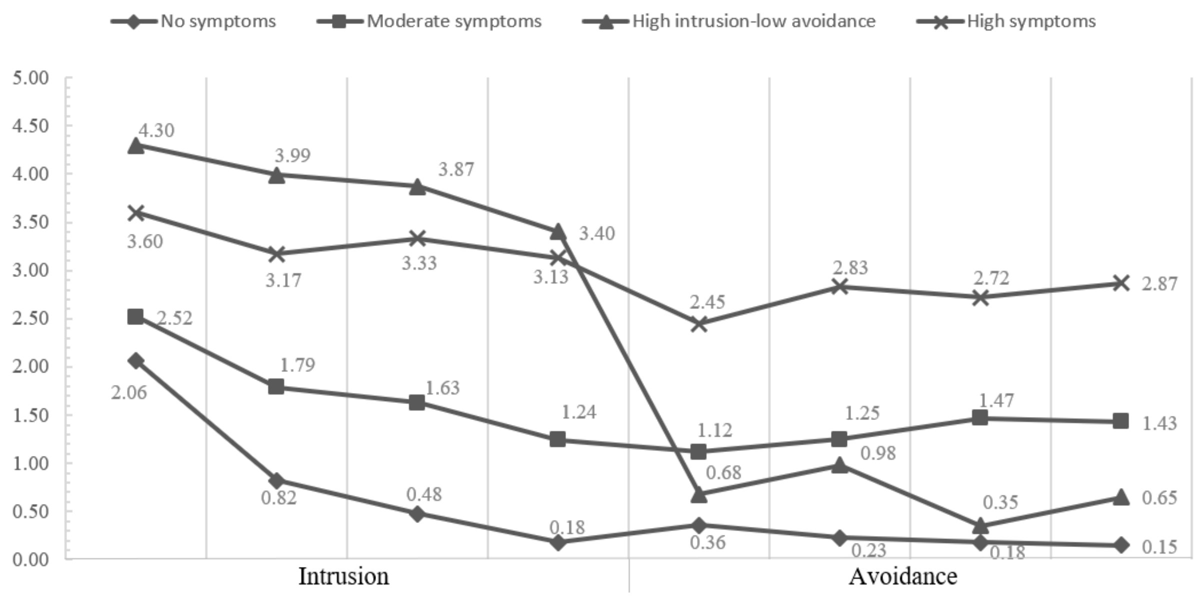

Figure S1. *Standardized means of the intrusion and avoidance across four profiles (N = 1,063)*
